# Analyzing Performance of YOLOx for Detecting Vehicles in Bad Weather Conditions

**DOI:** 10.3390/s24020522

**Published:** 2024-01-14

**Authors:** Imran Ashraf, Soojung Hur, Gunzung Kim, Yongwan Park

**Affiliations:** 1 Department of Information and Communication Engineering, Yeungnam University, Gyeongsan 38541, Republic of Korea; imranashraf@ynu.ac.kr (I.A.); sjheo@ynu.ac.kr (S.H.); 2 Institute of Information and Communication, Yeungnam University, Gyeongsan 38541, Republic of Korea; gzkim@yu.ac.kr

**Keywords:** autonomous vehicles, image processing, vehicle detection, YOLOx, deep learning

## Abstract

Recent advancements in computer vision technology, developments in sensors and sensor-collecting approaches, and the use of deep and transfer learning approaches have excelled in the development of autonomous vehicles. On-road vehicle detection has become a task of significant importance, especially due to exponentially increasing research on autonomous vehicles during the past few years. With high-end computing resources, a large number of deep learning models have been trained and tested for on-road vehicle detection recently. Vehicle detection may become a challenging process especially due to varying light and weather conditions like night, snow, sand, rain, foggy conditions, etc. In addition, vehicle detection should be fast enough to work in real time. This study investigates the use of the recent YOLO version, YOLOx, to detect vehicles in bad weather conditions including rain, fog, snow, and sandstorms. The model is tested on the publicly available benchmark dataset DAWN containing images containing four bad weather conditions, different illuminations, background, and number of vehicles in a frame. The efficacy of the model is evaluated in terms of precision, recall, and mAP. The results exhibit the better performance of YOLOx-s over YOLOx-m and YOLOx-l variants. YOLOx-s has 0.8983 and 0.8656 mAP for snow and sandstorms, respectively, while its mAP for rain and fog is 0.9509 and 0.9524, respectively. The performance of models is better for snow and foggy weather than rainy weather sandstorms. Further experiments indicate that enhancing image quality using multiscale retinex improves YOLOx performance.

## 1. Introduction

The last decade witnessed automation in several areas like industry, engineering, etc. The autonomous vehicle (AV) paradigm emerged as a potential solution to overcome the limitations of human drivers. In addition, AVs facilitate safe, comfortable, and eco-friendly future transportation. The current estimation of road accidents causing death is 1.3 million while the injuries are approximately 3.5, as stated by the World Health Organization [1]. Such accidents are predominantly caused by human error of judgment, slow reflexes due to intoxication, fatigue and sleep, and violations [2,3]. The prime objective of AVs is to avoid such errors and violations and eradicate or mitigate the chances of accidents, the final goal is to replace the human driver completely. The AV paradigm targets to reduce these accidents gradually with different levels of automation which are defined by the Society of Automotive Engineers (SAE). With each level of automation, the objective is to enable automation to reduce human errors and mitigate the probability of accidents. Finally, with full automation (level 5 of SAE), humans will be fully replaced and AVs will make all decisions. Test drives are now underway for level 4 (high automation) by different companies such as Tesla, Waymo, Audi, etc.

AVs are equipped with a large range and variety of sensors, both in terms of hardware and function. Such embedded sensors collect data for the surrounding environment continuously. The data collected by these sensors are later used for decision making, employing artificial intelligence (AI)-based frameworks. Radars, light detection and ranging (LiDAR), video cameras, infrared sensors, and thermal cameras are the commonly used AV sensors. The video camera provides continuous streams of traffic environment and is the common choice for environment perception during the daytime. Vehicle detection using video data is an important task for AVs for drivable space determining, path planning, traffic flow, vehicle counting, etc. However, vehicle detection using video data may become very challenging due to varying traffic environments where multiple vehicles and multiple categories of vehicles are to be detected. Even worse, bad weather conditions like snow, haze, and sandstorms make vehicle detection much more challenging.

Two desirable functions of a vehicle detection approach are its capacity to operate in real time and its ability to perform detection in illumination changing and bad weather conditions [4]. Several studies have presented the approaches to vehicle detection in different weather conditions [5]. Such studies utilize different kinds of information for vehicle detection, such as vehicle shadow [6], taillight [7], edge and color information [8,9], symmetry [10], etc. For vehicle detection, different machine learning and deep learning models are adopted. Machine learning models require feature extraction; consequently, a large variety of features has been investigated such as HOG [11], DPM [12], Haar or Haar-like [13], wavelet features, SURF [14], LBP, PCA, SIFT [15] features, etc. These features are investigated with different machine learning models like AdaBoost, k nearest neighbor, decision tree, etc. However, the support vector machine model is reported to produce better results.

Several pre-trained deep learning models have been introduced during the past few years to reduce the training requirements for vision tasks such as VGG16, ResNet, R-CNN, etc. The You Look Only Once (YOLO) series is one of the widely adopted models for object detection and has been well investigated in the literature. The YOLOx series is relatively new and this study considers it in the context of bad weather conditions. This study makes the following contributions:

- YOLOx has not been extensively studied, especially in the context of vehicle detection in bad weather conditions like snow and sandstorms. This study adopts three variants of YOLOx such as x, m, and l variants for performance analysis in rainy, foggy, sandstorm, and snowy weather conditions.
- Two strategies are adopted for experiments: using the original weather-affected dataset and using enhanced images. For image enhancement, a multiscale retinex approach is used in this study.
- For reproducibility, experiments involving using a publicly available dataset DAWN. Performance comparison is carried out using precision, recall, mean average precision, etc., as well as, comparing the performance of YOLOx with existing studies.

The rest of the paper is organized into four sections. Section 2 presents the related work. The methodology is presented in Section 3 while the experimental results and discussions are presented in Section 4. In the end, Section 5 concludes this study.

## 2. Related Work

Camera-based vehicle detection faces several challenges including varying sizes of vehicles, type of vehicle, change in color, etc. However, the most desirable functions of a vehicle detection system are real-time operation and detection in changing illumination [16]. These features are investigated with different machine learning models like AdaBoost, K nearest neighbor (KNN), decision tree (DT), etc.

Recently, the use of deep learning models in image processing tasks showed superb results [17]. As a result, several studies embrace deep learning models for improving vehicle detection accuracy. These models are broadly categorized into two groups: target-level and semantic segmentation-level vehicle detection. Where the former uses the label assigned to the whole vehicle while the latter uses label assignment to each pixel of a particular target in the image. While both approaches have favorable results, semantic segmentation is reported to show superior results [18]. The authors present a deep learning framework for vehicle detection and tracking in adverse weather conditions in [19]. Images from video streams are first adjusted using illumination, reflection, and weighted fusion. Vehicle detection is carried out using the preprocessed image with the YOLOv3 model. Superior results are reported using YOLOv3 compared to existing models for bad weather vehicle detection.

Along the same lines, in [20], a real-time vehicle detection system is designed for bad weather conditions. A visibility complementary approach is proposed to correct images for obtaining improved detection results. The proposed image enhancement approach aims at removing the impact of raindrops. The corrected images are then used with YOLOv3 for vehicle detection. Experiments involve using rainy weather, gare, and haze conditions. The results report 87.27% positive detection for rainy weather and 89.2% and 79.21% positive detection rates for glare and haze, respectively. The authors adopt a faster R-CNN in [21] for vehicle detection in rainy conditions. The faster R-CNN is employed along with region proposal networks (RPNs) for improved vehicle detection. To detect vehicles of varying sizes, several RPNs are used with different sizes to enhance detection performance. Experimental results on different datasets indicate 95.16% average precision on the LISA dataset.

Real-time vehicle detection is one of the basic requirements of AV; however, camera-based vehicle detection is computationally complex, requiring high on-board processing capabilities. For this reason, a trade-off is often made between accuracy and speed [22]. Sensor fusion is recommended to increase the detection performance of a framework where the data from the camera can be merged with radar, light detection and ranging (LiDAR), etc., to obtain better detection results. The authors present a multi-sensor fusion approach to improve vehicle detection in bad weather [23]. A LightGBM model is used for vehicle target extraction using radar. The region of interest (ROI) is estimated using infrared images concerning the distribution of radar targets. Later, Haar-like features are utilized to enhance vehicle detection. An accuracy of 92.4% is reported in bad weather conditions.

Vehicle detection in complex environments like traffic jams and varying weather conditions is carried out in [24]. The authors specifically consider different illumination conditions like sunrise and sunset time, as well as, cloudy days, rainy days, sunny days, etc. The authors design an adaptive vehicle detector for vehicle detection in bad weather without extracting background information. For removing the effect of weather and light, histogram extension is utilized. Similarly, for extracting moving objects, the grey-level differential value is leveraged. Experimental results indicate that the proposed approach is both robust and accurate, especially for complex scenarios. Similarly, the study [25] proposes an approach for vehicle detection in sandstorms using videos collected from YouTube for sandstorms in different countries including Kuwait, Saudi Arabia, Arizona, etc. The study also utilized the Traffic-Net dataset which contains videos of burning vehicles due to accidents and other complex situations. Promising results are reported in the study.

The study [26] introduces multi-scale vehicle detection for different weather conditions using the YOLOv4 model. The research focuses on designing a single approach for vehicle detection in dust, sand, snow, and rain for day and night time traffic alike. The CSPDarnet53 is used as the baseline with spatial pyramid pooling for vehicle detection. Experiments involve using the DAWN dataset, as well as, the augmented DAWN dataset with hue, saturation, noise, blur, etc. A mean average precision of 81% is reported. Similarly, an enhanced YOLO model is proposed in [27] for vehicle detection in foggy weather. For better results, a dehazing module is added to YOLO. The dehazing module is based on multi-scale retinex with color restoration. Experiments are performed using the BDD100K dataset, as well as, fogged images. The results show that the detection accuracy varies with the degree of foggy weather.

A pedestrian and vehicle detection algorithm is presented in [28] that utilizes the Swin Transformer. An end-to-end vehicle detection algorithm, PVformer is proposed for enhanced vehicle and pedestrian detection accuracy. It comprises a deraining module that mitigates the impact of rain on images and enhances detection performance. Experiments involve using multiple datasets including Rain100L, Rain100H, Raindrop, and Attentive GAN-Data. Experimental results show an mAP of 88.9%.

Feature extraction is a key and critical part of object detection frameworks and an appropriate feature selection approach can potentially impact their performance. The study [29] focuses on the adoption of a pixel-level supervision neural network for obtaining appropriate and discriminating features to improve low detection rates and reduce high false alarms. Experimental results indicate improved performance of the proposed approach with only a 3.02% false alarm rate.

The authors present a novel approach in [30] for vehicle detection in snowy conditions, along with the introduction of a real-world object detection dataset. The proposed approach is an unsupervised training strategy that performs a quantitative evaluation of snow on various objects. In addition, a lightweight network is built using YOLOv5s for object detection in snowy conditions. The results demonstrate that the proposed CF-YOLO shows higher confidence for vehicle detection and reduces false detection. The model shows better performance, particularly for difficult scenarios, where vehicle detection is hard. An average precision of 71.10% is obtained with less number of epochs by the proposed approach.

The study [31] focuses on alleviating the problems of image enhancement and incorporating the latent information in object detection tasks in bad weather conditions. A novel approach, image adaptive YOLO (IA-YOLO) is proposed where each image is improved adaptively. The authors introduce a differentiable image processing for bad weather. Experiments involve the use of YOLOv3, DSNet, DAYOLO, and ZeroDCE in comparison to the proposed approach. The results show a 70.02% mAP for the foggy dataset.

Despite the adoption of YOLOV variants in the existing literature, the YOLOx model is not investigated well for vehicle detection in bad weather conditions. This study employs YOLOx for vehicle detection in rain, fog, snow, and sandstorms to analyze its performance.

## 3. Methodology

**Figure 1:**
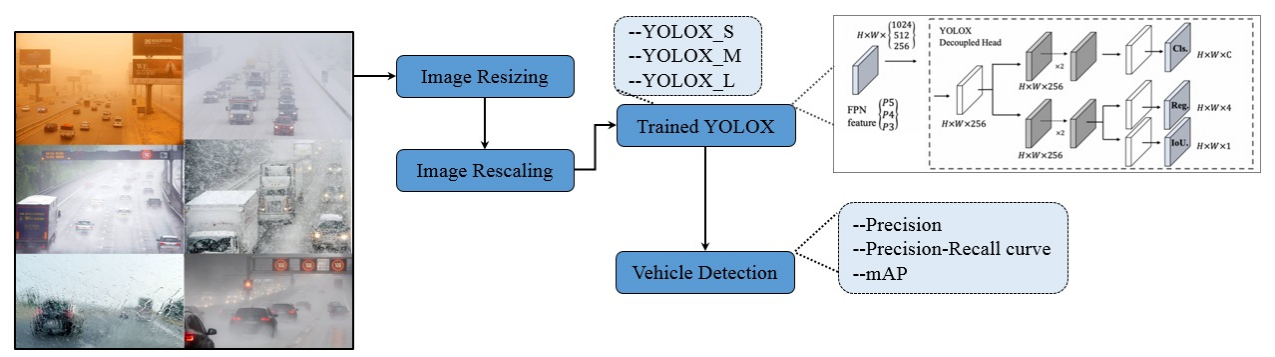
Architecture of the adopted methodology for vehicle detection.

This study uses the YOLOx model for vehicle detection in snow and sandstorms where the visibility and illumination conditions are low and investigates the efficiency of three variants of YOLOx. Figure 1 shows the workflow of the adopted methodology for vehicle detection using YOLOx.

### 3.1. YOLOx

YOLOx is an improvement in the YOLO series and is anchor-free. Decoupled head and leading assignment strategies are also adopted [32]. YOLOv3, which is widely used for vehicle detection, and YOLOv4 and YOLOv5 are anchor-based approaches. The anchor-free detectors of YOLOx reduce the number of design parameters while decoupled head improves convergence speed. Training time is improved by SimOTA advanced label assignment. This study utilizes the pre-trained models that can perform classification on 80 different classes.

### 3.2. Dataset

For experiments, the publicly available dataset DAWN is used [33]. The DAWN dataset contains 1000 images from real traffic conditions in different weather conditions. It contains images of rain, fog, snow, and sandstorms. Data collection scenarios involve urban, highway, and freeway and contain single to multiple vehicles in a single image. Four types of vehicles are present in the dataset including cars (the predominant class), motorcycles, buses, and trucks. For each scenario, the number of images is different, as shown in Table 1.

### 3.3. Vehicle Detection

The methodology adopted for vehicle detection in sand and snow storms is shown in Figure 1. The images for snow, rain, fog, and sandstorms are input for preprocessing. The collected images have different dimensions, so image resizing is performed to transform the image into 640 × 640 dimensions, and image rescaling is carried out to maintain the height-to-width ratio. The processed images are then fed into a pre-trained YOLOx model for vehicle detection. The model’s performance is analyzed in terms of mean average precision and precision–recall curve.

## 4. Results and Discussions

This section discusses the results of the ‘s’, ‘m’, and ‘l’ variants of the YOLOx series for vehicle detection in two bad weather conditions including rain and snow.

### 4.1. Experimental Setup

Experiments are performed on an Intel Core i7 machine running on a Windows 10 operating system and 16 GB RAM. Matlab 2022b is used for model implementation. The number of snow- and sandstorm-affected images used for model testing is different.

### 4.2. Results for Snow

Table 2 shows the precision results of all three variants of YOLOx for vehicle detection under snowstorm conditions. The precision is calculated for each class in the dataset. True positives are calculated using an intersection over union (IoU), and true positives are those with an IoU ≥ 0.5.

**Figure 2:**
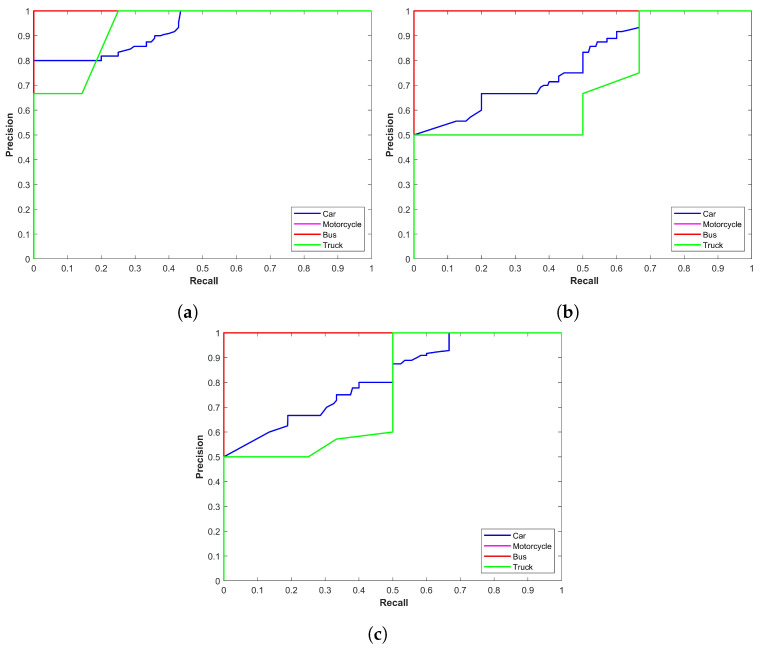
Precision–recall curve for YOLOx in snow storms, (**a**) YOLOx-s, (**b**) YOLOx-m, and (**c**) YOLOx-l.

Figure 2 illustrates the precision–recall curve for YOLOx models for snow storms. The plot indicates that the performance of YOLOx-s is better compared to other variants concerning car, motorcycle, and truck detection. On the other hand, YOLOx-m shows better performance for bus detection. YOLOx-l has marginally lower precision for truck and car detection than YOLOx-s and YOLOx-m, respectively. The main emphasis is on the precision–recall curve of the ‘car’ class as it has the highest number of images. Precision for the YOLOx-s is 0.9588 for the car class which is better than 0.9167 and 0.9317 from YOLOx-m and YOLOx-l models, respectively.

### 4.3. Results for Sand Storms

**Figure 3:**
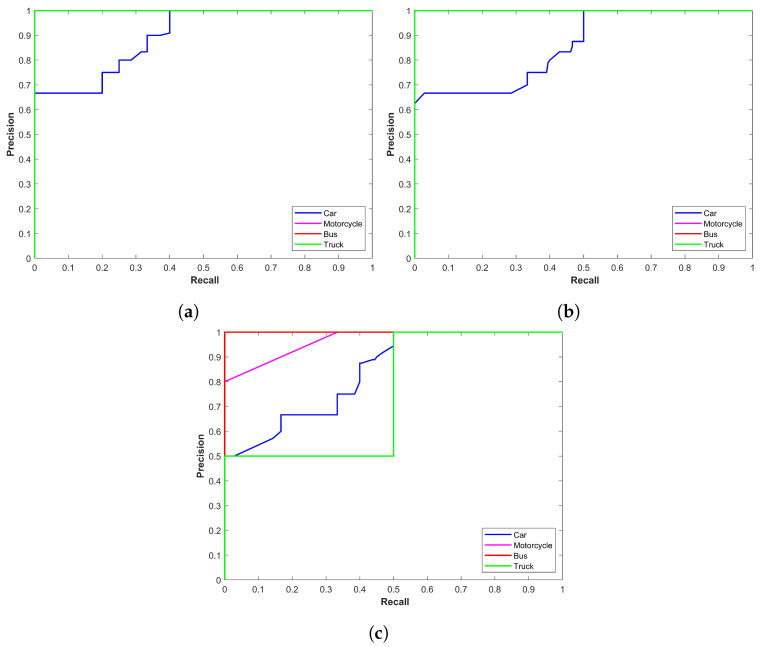
Precision–recall curve for YOLOx in sandstorms, (**a**) YOLOx-s, (**b**) YOLOx-m, and (**c**) YOLOx-l.

Class-wise precision results for the YOLOx model in sandstorms are given in Table 3. In contrast to results for snow conditions, where YOLOx-s performed better for car detection, YOLOX-l shows better performance for car detection in sandstorms with a 0.8543 precision compared to 0.8526 and 0.8501 from YOLOx-m and YOLOx-s, respectively. However, for motorcycle, bus, and truck detection in sandstorms, the precision scores for YOLOx-s are better. Figure 3 shows precision–recall curves for all models in sandstorms. It shows the mixed performance of models for different classes in the case of vehicle detection in sandstorms.

### 4.4. Results for Rainy Conditions

Table 4 shows the results of all three variants of YOLOx for vehicle detection under bad weather conditions concerning precision and average recall. Average precision and average recall are calculated for all images concerning vehicle detection. True positives are calculated using an intersection over union (IoU) indicating that the prediction is correct if IoU ≥ 0.5.

**Figure 4:**
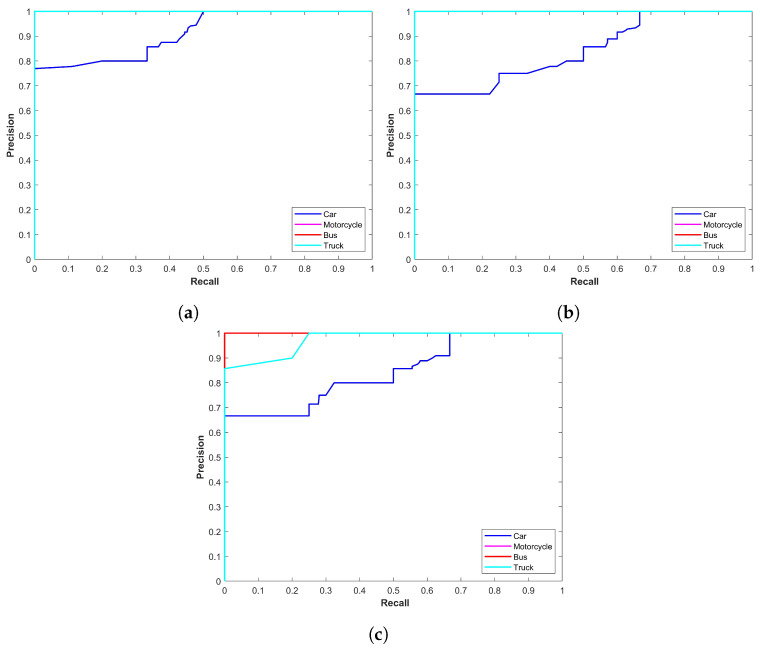
Precision–recall curves for YOLOx-s for rainy conditions, (**a**) YOLOx-s, (**b**) YOLOx-m, and (**c**) YOLOx-l.

Table 4 shows the performance of YOLOx for rainy conditions where class-wise precision is reported. Figure 4 shows the precision–recall curve for YOLOx models for rainy conditions; Figure 4a–c show the precision–recall curves for YOLOx-s, YOLOx-m, and YOLOx-l, respectively. It shows that the performance of YOLOx-s is better compared to other variants. The main emphasis is on the precision–recall curve of the ‘car’ class as it has the highest number of samples in the dataset. Precision for the YOLOx-s is 0.9283 for the car class, which is better than 0.9090 and 0.9124 for the YOLOx-m and YOLOx-l models, respectively. Motorcycle objects are few and they can have high precision and better precision–recall curves.

### 4.5. Results for Foggy Conditions

**Figure 5:**
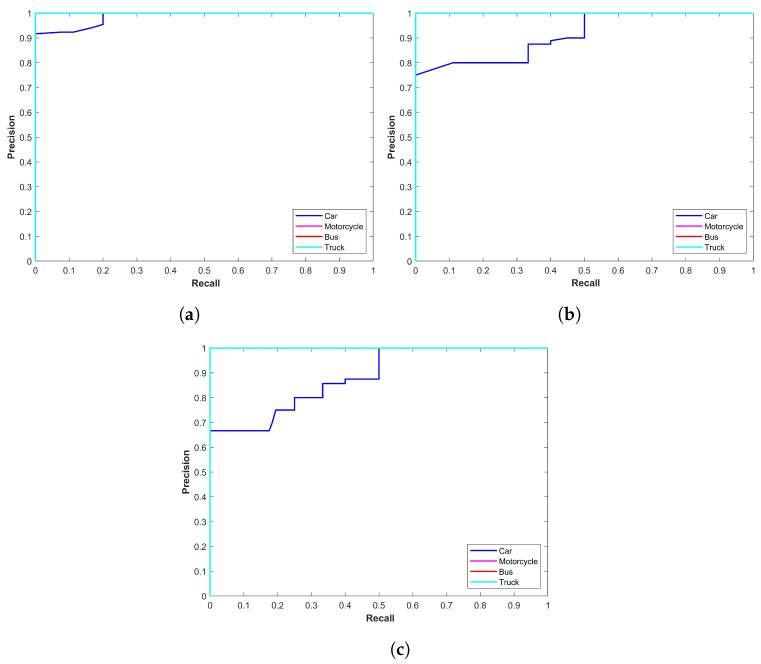
Precision-recall curve for YOLOx in foggy conditions, (**a**) Curve for YOLOx-s model, (**b**) Curve for YOLOx-m model, and (**c**) Curve for YOLOx-l model.

Results for YOLOx mode in foggy weather conditions are given in Table 5. YOLOx-s again indicates a better precision score of 0.9617 than other variants for the car class. The same is true for other classes as well. Figure 5 shows precision–recall curves for all models in foggy conditions. It indicates better performance of YOLOx-s compared to other models.

### 4.6. Results Regarding Mean Average Precision

Besides the precision and precision–recall curve, the mAP metric is a widely used performance evaluation metric for object detection. It is especially important when the detection task involves multi-class detection. This study also uses mAP which is calculated using

(1) $$mAP=\frac{1}{N}\sum_{i=1}^{N}AP_{i}$$

where *N* indicates the total number of classes, four in this case, and $AP_{i}$ indicates the average precision of class *i*.

Table 6 shows mAP for all models for rain, fog, sand, and snow storms, demonstrating the better performance of the YOLOx-s model over other models.

For snowstorm weather, the performance of YOLOx-s is marginally better with an mAP of 0.8983 than YOLOx-m, which has an mAP of 0.8930. However, for sandstorms, it shows much better performance with 0.8656 mAP compared to 0.8476 and 0.8130 mAP scores of YOLOx-m and YOLOx-l, respectively. Similarly, YOLOx-s shows superior performance for rainy and foggy conditions with 0.9509 and 0.9524 mAP which is better than other variants.

**Figure 6:**
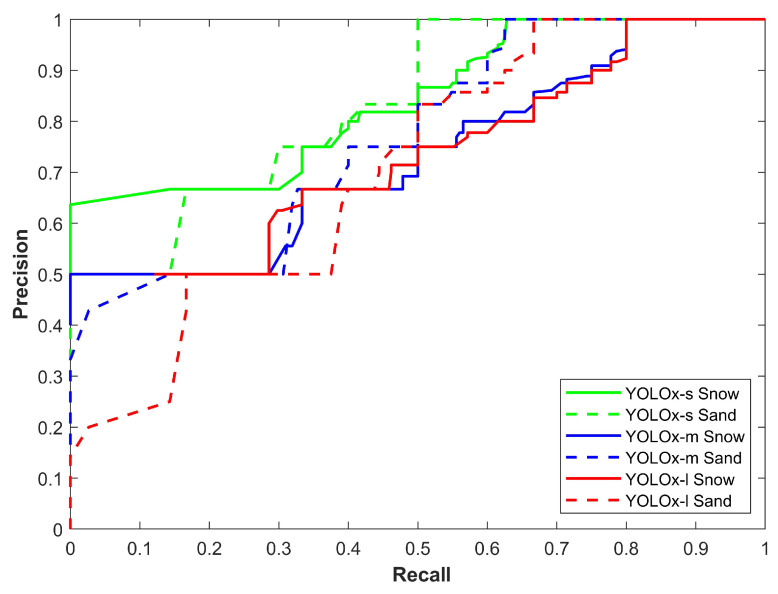
Precision–recall curve of all models for all classes in snow and sand.

Figure 6 demonstrates the precision–recall curve of all YOLOx variants for sand and snow storms. Two important observations are the change in the performance of models in snow and sandstorms and the difference in models’ individual performance. First, the YOLOx-s model proves to be more precise with a better precision–recall curve while the second is the better performance of models in snow storms. Figure 6 shows that models perform poorly in sandstorms.

**Figure 7:**
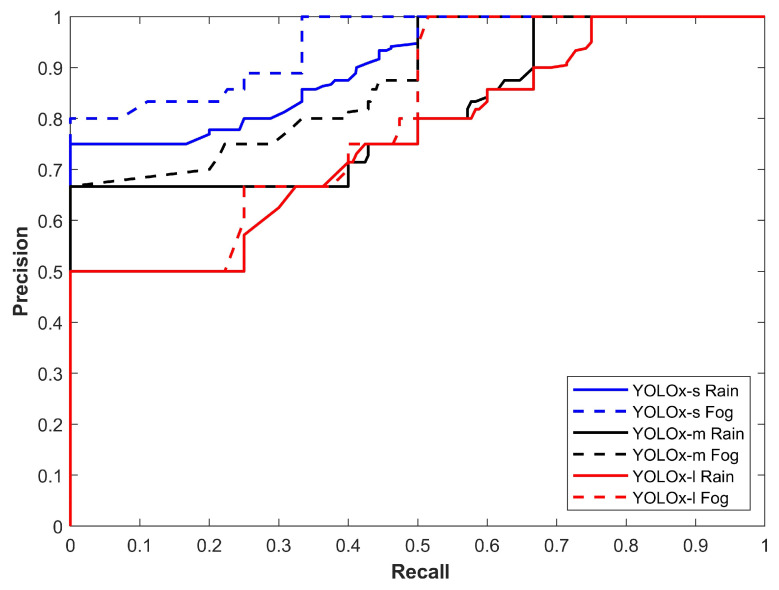
Precision–recall curve of all models for all classes in rain and fog.

Figure 7 illustrates the precision–recall curve of all YOLOx variants for rain and foggy weather. It shows two noteworthy points: the first is the better performance of the YOLOx-s model for object detection while the second is the better performance in foggy conditions. The results displayed in Figure 7 show that models perform poorly in rainy conditions.

Table 7 shows the results regarding the number of ground truth objects and detected objects by each model for snow and sandstorms. As stated earlier, the images predominantly contain the ‘car’ class, followed by truck, bus, and motorcycle. The results indicate that the YOLOx-m and YOLOx-l detect a higher number of objects than the YOLOx-s model. However, these detections contain many false positive samples which degrade their overall performance.

Results regarding the number of ground truth objects and detected objects by each model for rainy and foggy conditions are provided in Table 8. As stated earlier, predominantly the images contain the ‘car’ class, followed by truck, bus, and motorcycle. The results indicate that the YOLOx-m and YOLOx-l detect a higher number of objects than the YOLOx-s model. However, these detections contain many false positive samples which degrade their overall performance.

### 4.7. Performance of YOLO with Enhanced Images

**Figure 8:**
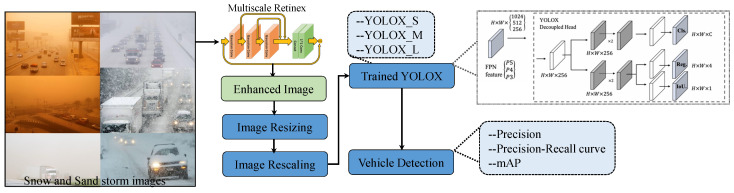
Architecture of the methodology involving image enhancement for vehicle detection.

The images in the used dataset contain noise introduced by weather conditions like snow, rain, etc., which affects the performance of YOLO. For improving the performance of the model, image enhancement is carried out before feeding it to the YOLO, as shown in Figure 8. In addition to the steps followed for vehicle detection using YOLO, an image enhancement strategy is adopted to improve the image quality. This step involves enhancing image quality using color restoration by multiscale retinex (MSR) adopted from [34].

Contrary to single-scale retinex, which requires a trade-off between range compression and color rendition, MSR affords a better trade-off between local dynamic range and color. The following equation is used in MSR

(2) $$R_{MSR_{i}}=\sum_{n=1}^{N}w_{n}R_{n_{i}}=\sum_{n=1}^{N}\left[logI_{i}\left(x,y\right)-long\left(F_{n}\left(x,y\right)*I_{i}\left(x,y\right)\right)\right]$$

where *N*, and $w_{n}$ show the number of scales and their weight, respectively, while $F_{n}\left(x,y\right)=C_{n}exp\left[-\left(x^{2}+y^{2}\right)/2\sigma_{n}^{2}\right]$.

**Figure 9:**
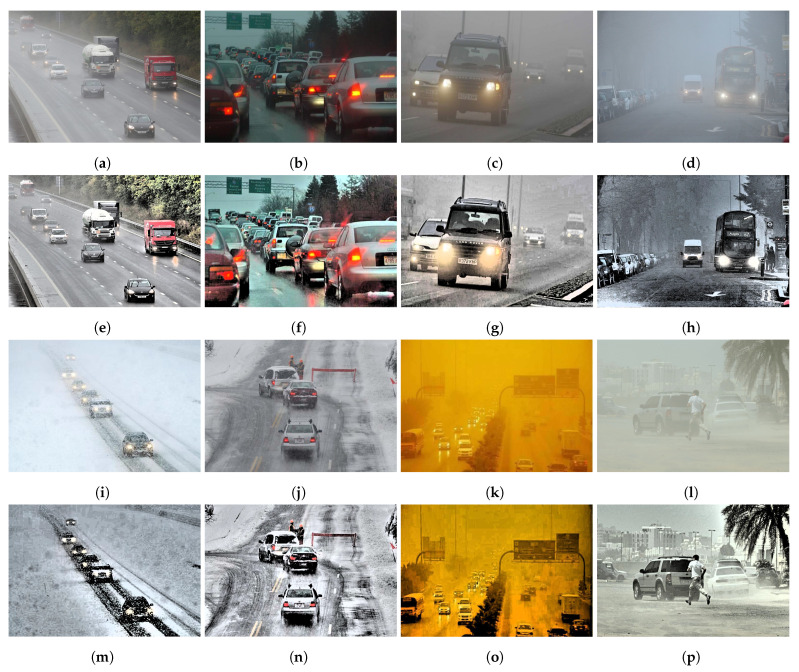
Original and enhanced images using multi scale retinex, (**a**,**b**) rain-affected, (**c**,**d**) fog-affected, (**e**–**h**) enhanced images for rain and fog, (**i**,**j**) snow-affected, (**k**,**l**) sand-affected, and (**m**–**p**) enhanced images for snow and sand.

MSR image enhancement helps improve light and color transformation which is expected to improve the performance of YOLO vehicle detection. A few images before and after restoration are presented in Figure 9.

**Figure 10:**
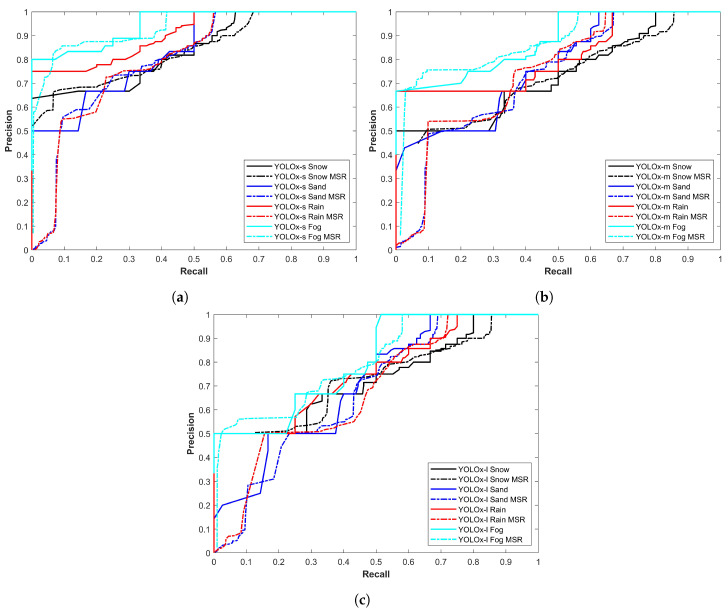
Precision–recall curves for YOLO variants using enhanced images, (**a**) YOLOx-s model performance for all classes, (**b**) YOLOx-m model performance for all classes, and (**c**) YOLOx-l model performance for all classes.

After improving the image quality, the same procedure is followed for vehicle detection as was carried out for vehicle detection from the original images using YOLOx. Precision recall curves for all variants of the YOLOx model are presented here in comparison to the curves for the original images. Figure 10 shows a performance comparison of YOLO variants before and after image enhancement is carried out. The results are indicative of improved performance thereby showing the potential of image enhancement to improve the vehicle detection performance of the YOLO model.

### 4.8. Speed and Floating Point Operations per Second

Speed and floating point operations per second (FLOPs) are regarded as important parameters to evaluate computer performance and are considered a better measure compared to instructions per second. Table 9 shows the speed, number of parameters, and GFLOPs of the three YOLO variants used in this study.

### 4.9. Performance Comparison with Existing Studies

A comparative analysis with existing studies is also carried out to evaluate the efficacy of YOLOx in relation to other variants. Table 10 shows the comparison of the [30,31] in the context of YOLOx variants for foggy conditions. The results show that YOLOx performs better. However, it must be noted that the CF-YOLO model is tested in hard foggy conditions where vehicle detection is difficult in comparison to light or medium fog conditions which are adopted in [31].

### 4.10. Discussions

This study leverages the YOLOx model, the latest addition in the YOLO series, for vehicle detection in sand and snow storms. During experiments, several important points are observed which are discussed here.

**Figure 11:**
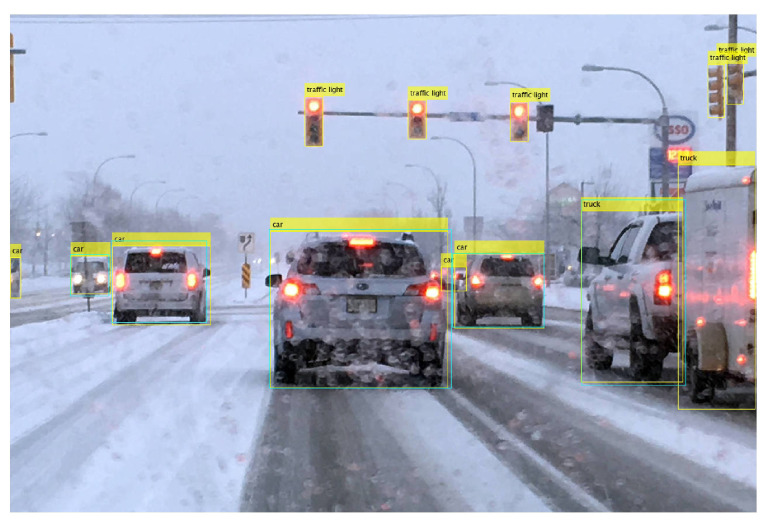
Car-carrying trailer detected as ‘truck’ in snow.

The first problem originates from car-carrying trailers. As shown in Figure 11, YOLOx treats both as different vehicles and detects them separately. At the same time, the car carrying the trailer is also detected as a ‘truck’ which is a false positive. The yellow rectangle indicates YOLOx detection while the cyan rectangle shows the ground truths.

**Figure 12:**
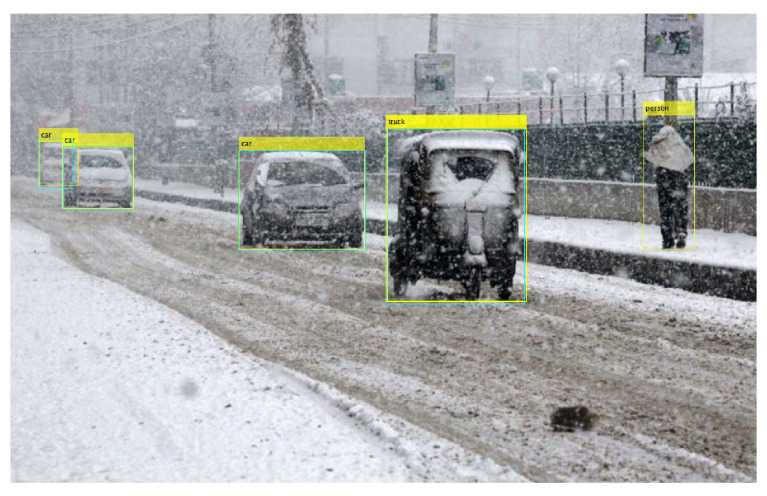
A sample of false positive from YOLOx in snow.

Secondly, another type of false positive from YOLOx models comes in the form of a ‘rickshaw’ classified as a ‘truck’. One sample of such false positives is shown in Figure 12 where the rickshaw is labeled as the truck.

**Figure 13:**
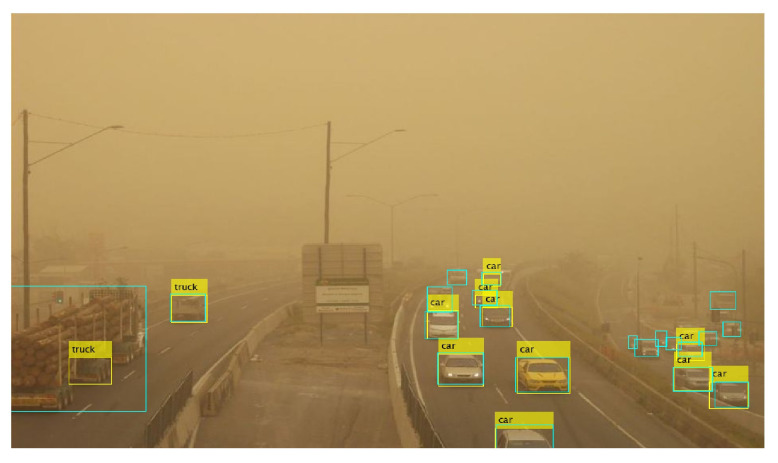
Wrong detections from YOLOx in a sandstorm.

Thirdly, several cases are observed where the YOLOx model detects a partial part of a vehicle as the vehicle which means that the boundary identification is not proper. For example, Figure 13 shows one such instance where the wheel part of the truck is identified as the truck by the model.

**Figure 14:**
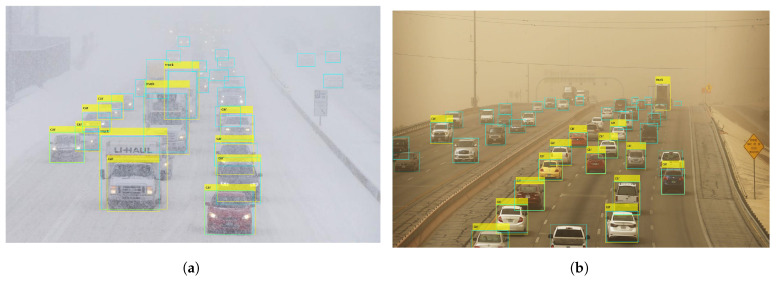
Missed vehicle from YOLOx in bad weather, (**a**) snow, and (**b**) sandstorm.

Finally, the performance of YOLOx for vehicle detection is affected primarily on account of the higher number of vehicles in a scene rather than sand or snow storms. For example, Figure 14a shows detection in rainy conditions where the road is covered by snow and moving traffic is affecting visibility. It can be observed that several vehicles are not detected by the model.

Similarly, Figure 14b shows a large number of vehicles in sandstorms. It can be seen that the visibility is better compared to Figure 14a. However, the model misses more than 15 vehicles on the scene. The model specifically shows inefficacy in detecting partially occluded and partial vehicles. It emphasizes the need for image correction and enhancement approaches to improve the detection performance of the YOLOx model.

**Figure 15:**
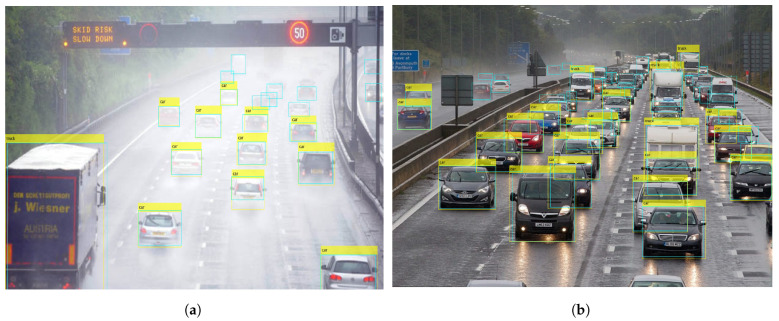
Detections from YOLOx in bad weather, (**a**) Wrong detections in medium rain, and (**b**) Wrong detections in light rain.

Experiments involving rainy and foggy conditions, also highlight several problems of the YOLOx model for vehicle detection. The first is the pattern of vehicle detection by YOLOx which is affected mostly on account of the higher number of vehicles in a scene rather than weather conditions. Figure 15a shows detection in rainy conditions where the road is covered by water and moving traffic is affecting the visibility. The yellow rectangle indicates YOLOx detection while the cyan rectangle shows the ground truths. It can be observed that nine vehicles are missed by the model. In Figure 15b, the visibility is better; however, the number of vehicles is higher. In this case, as well, the model misses more than 15 vehicles. The model shows inefficacy in detecting partially occluded and partial vehicles.

**Figure 16:**
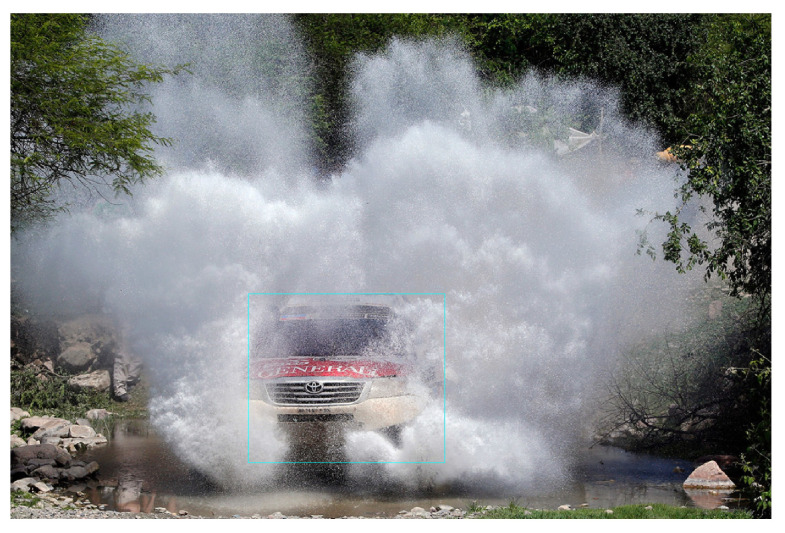
Missed vehicle from YOLOx in bad weather.

Secondly, some obvious vehicles are missed by the model. Let us have a look at Figure 16, which contains only one vehicle. Due to the water splash, the vehicle is occluded and the model is unable to detect the vehicle, although it is very visible.

**Figure 17:**
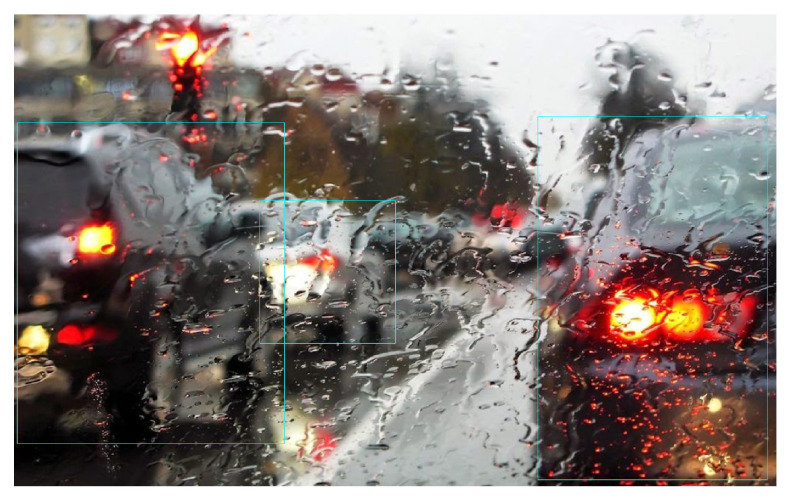
No detection in screen covered by rainwater.

Figure 17 shows another case of vehicle detection affected by the rain. The cases where the windshield is partially blocked by the rainwater lead to no vehicle detection. The visibility is severely affected and vehicles are not visible except for the tail lights. YOLOx shows poor performance and can not detect vehicles. It emphasizes the need for image correction and enhancement approaches to improve the detection performance of the YOLOx model.

**Figure 18:**
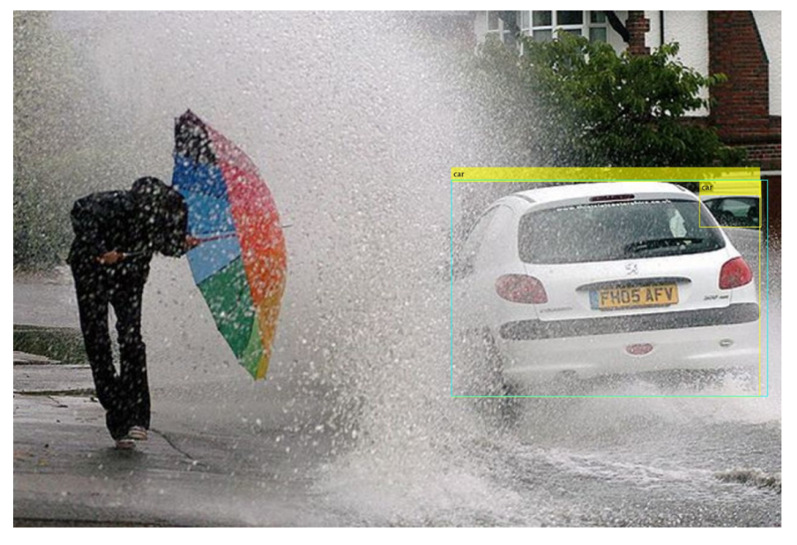
YOLOx car detection in window glass.

Finally, a few samples are observed where YOLOx detects vehicles that are not in the ground truth data. For example, Figure 18 shows one such instance where a car is detected by the YOLOx. The detected car is a reflection of an on-road car in the window of a house.

YOLO model has shown promising results for object detection and has been considered more efficient than CNN, Faster R-CNN, and similar other models. YOLO is proven to be more efficient for object detection due to its end-to-end training. It provides more robust and accurate results. However, weather-affected images contain noise in the form of rain and fog drops or sand grains, etc., which results in poor image quality. It is observed that the results of the YOLOx variant are bad when there is less contrast and poor light conditions. Poor light conditions also lead to poor contrast which affects the model’s performance for vehicle detection.

## 5. Conclusions

The objective of this study is to analyze the efficacy of the YOLOx model for vehicle detection in bad weather conditions, particularly rainy and foggy conditions, and snow and snow storms. For experiments, the publicly available benchmark dataset DAWN is used, and ‘s’, ‘m’, and ‘l’ variants of YOLOx are utilized. The results show that YOLOx often shows better performance for different classes of vehicles than its counterparts. It has a 0.8983 mAP for snowy conditions and achieves a 0.8656 mAP for sandstorms. Similarly, experimental results for rainy and foggy conditions demonstrate a better performance of YOLOx-s over the other two models with an mAP of 0.9509 in rain and 0.9524 in foggy conditions. Overall, the models show better performance in snow storms than in sandstorms. All models tend to perform better in fog than in rain. It is also observed that the performance of YOLOx is degraded when the image has a higher number of vehicles, partially occluded vehicles, and low visibility indicating the scope of image enhancement approaches for better performance. The model experienced degraded results for poor light conditions where the contrast is low; we intend to perform further experiments with image enhancement approaches. Moreover, the categorization of weather conditions into light, medium, and hard should also be taken into account as weather intensity has a huge impact on YOLO performance. Performance comparison in the context of other models like Faster CNN, etc., will also be considered.

## Figures and Tables

**Table 1:** Images for each category in the dataset.

Scenario	# of Images	Classes
Snow	205	Car, motorcycle, bus and truck
Sand	324	Car, motorcycle, bus and truck
Rain	200	Car, motorcycle, bus and truck
Fog	300	Car, motorcycle, bus and truck

**Table 2:** Results for class-wise precision of YOLOx for snow.

Model	Precision			
	Car	Motorcycle	Bus	Truck
YOLOx-s	0.9588	1.00	0.8646	0.7698
YOLOx-m	0.9167	1.00	0.9167	0.7385
YOLOx-l	0.9317	0.50	0.9062	0.7600

**Table 3:** Precision and recall of YOLOx series for sandstorms.

Model	Precision			
	Car	Motorcycle	Bus	Truck
YOLOx-s	0.8501	0.8333	1.00	0.7788
YOLOx-m	0.8526	0.8269	0.9737	0.7373
YOLOx-l	0.8543	0.8083	0.9408	0.6488

**Table 4:** Class-wise precision and recall of YOLOx series for rain conditions.

Model	Precision			
	Car	Motorcycle	Bus	Truck
YOLOx-s	0.9283	1.00	1.00	0.8752
YOLOx-m	0.9090	1.00	1.00	0.8562
YOLOx-l	0.9124	0.6667	0.8846	0.8223

**Table 5:** Class-wise precision and recall of YOLOx series for fog conditions.

Model	Precision			
	Car	Motorcycle	Bus	Truck
YOLOx-s	0.9617	1.00	1.00	0.8479
YOLOx-m	0.9534	1.00	0.9872	0.8167
YOLOx-s	0.9485	1.00	1.00	0.8117

**Table 6:** Experimental results for YOLOx concerning mAP.

Model	mAP			
	Snow	Sand	Rain	Fog
YOLOx-s	0.8983	0.8656	0.9509	0.9524
YOLOx-m	0.8930	0.8476	0.9413	0.9393
YOLOx-l	0.7745	0.8130	0.8215	0.9400

**Table 7:** Results for original and detected vehicles from YOLOx.

Model	Car		Motorcycle		Bus		Truck		All Classes	
	Total	Detected	Total	Detected	Total	Detected	Total	Detected	Total	Detected
**Snow**										
YOLOx-s	1751	1078	1	1	22	29	108	125	1882	1233
YOLOx-m	1751	1185	1	1	22	22	108	152	1882	1360
YOLOx-l	1751	1076	1	2	22	28	108	153	1882	1259
**Sand**										
YOLOx-s	977	641	18	12	35	17	119	99	1149	769
YOLOx-m	967	702	18	13	35	18	117	125	1137	858
YOLOx-l	977	683	18	13	35	22	119	139	1149	857

**Table 8:** Original and detected vehicles from YOLOx.

Model	Car		Motorcycle		Bus		Truck		All Classes	
	Total	Detected	Total	Detected	Total	Detected	Total	Detected	Total	Detected
**Rain**										
YOLOx-s	1355	903	4	2	13	8	188	105	1560	1018
YOLOx-m	1355	1002	4	2	13	16	188	137	1560	1157
YOLOx-l	1355	972	4	2	13	17	188	182	1560	1173
**Fog**										
YOLOx-s	1120	685	18	9	49	25	110	60	1297	779
YOLOx-m	1120	750	18	13	49	30	110	81	1297	874
YOLOx-l	1120	729	18	12	49	24	110	103	1297	868

**Table 9:** Speed, FLOPS, and number of parameters for YOLO models.

Variant	Speed (ms)	FLOPS (Mega)	Parameters (m)
YOLOx-s	9.9	0.4831	9.0
YOLOx-m	12.3	0.9834	25.3
YOLOx-l	14.5	1.2587	54.2

**Table 10:** Performance analysis of YOLOx concerning existing works.

Reference	Model	Weather Intensity	Weather	mAP
[30]	CF-YOLO	Hard	Foggy	71.10%
[31]	IA-YOLO	Medium	Foggy	70.02%
Current study	YOLOx-s	Light-to-Medium	Foggy	95.24%
Current study	YOLOx-m	Light-to-Medium	Foggy	93.93%
Current study	YOLOx-l	Light-to-Medium	Foggy	94.00%

## Data Availability

Data are contained within the article.
